# Functional dissection of the prototype foamy virus glycoprotein heparan sulfate binding site

**DOI:** 10.1186/s12977-026-00676-7

**Published:** 2026-03-21

**Authors:** Max Büttrich, Nicole Stanke-Scheffler, Thomas Calcraft, Peter B. Rosenthal, Dirk Lindemann

**Affiliations:** 1 Institute of Medical Microbiology and Virology, Faculty of Medicine “Carl Gustav Carus”, Technische Universität Dresden, Fetscherstr. 74, 01307 Dresden, Germany; 2 CRTD/DFG-Center for Regenerative Therapies Dresden, Technische Universität Dresden, Dresden, Germany; 3 Structural Biology of Cells and Viruses Laboratory, The Francis Crick Institute, London, NW1 1AT UK

**Keywords:** Foamy virus, Glycoprotein, Heparan sulfate, Attachment factor, Binding site

## Abstract

**Background:**

The foamy virus (FV) glycoprotein complex (GPC) facilitates exceptionally broad species and tissue tropism. While cell surface heparan sulfate (HS) serves as a known attachment factor, it is not essential for viral entry. Recent high-resolution structures of GPCs from various FV species identified an evolutionarily conserved, positively charged surface patch (PCSP) on the receptor-binding domain (RBD) as a putative HS-binding site (HSBS). To date, only the gorilla FV (SFVggo) HSBS has been functionally characterized, demonstrating the role of basic PCSP residues in HS-dependent attachment. Experimental evidence supporting a universal role for the GPC PCSP across other FV species is currently lacking.

**Results:**

The prototype FV (PFV) GPC PCSP consists of four central residues surrounded by five peripheral, positively charged residues. Using charge-switch mutagenesis, we investigated the functional role of eight PCSP residues. The central residues—K_343_, K_355_, R_357_, and K_368_—proved essential for HS-dependent attachment and infection across various target cells. Individual mutations of these residues reduced attachment and infectivity in HT1080 cells by 50- to 100-fold. Among peripheral residues, only K_356_ contributed significantly to these processes on different HS-expressing target cells. Notably, all mutant PFV GPCs maintained levels of attachment and infectivity in HS-deficient cells similar to those of the wild-type, though these levels were 10- to 30-fold lower than in HS-expressing parental cells but well above background.

**Conclusions:**

The minimal HSBS of the PFV GPC is defined by four central, evolutionarily conserved positively charged residues. Substituting these with negatively charged amino acids abolishes HS-dependent attachment and severely reduces specific infectivity. The minor impact of the peripheral residue mutation K_356_E, combined with the lack of evolutionary conservation among most peripheral positively charged residues in primate FV species, suggests these residues play only a secondary role in HS interaction. Furthermore, the residual infectivity of PCSP mutants in HS-deficient cells confirms that HS is an important attachment factor but not an essential entry receptor. The functional homology between PFV and SFVggo GPCs strongly suggests that this conserved PCSP constitutes a universal HS-binding site across all FV species.

**Supplementary Information:**

The online version contains supplementary material available at 10.1186/s12977-026-00676-7.

## Background

The plasma membrane represents the first barrier for viruses to overcome upon starting their replication in permissive and susceptible host cells. Crossing the plasma membrane barrier to release the viral capsid into the cytoplasm is a multistep process, which may include separate cell surface attachment and membrane fusion processes. Like many other viruses, simian (SFV) and feline (FFV) foamy viruses were reported to use heparan sulfate (HS) as an important attachment factor to greatly facilitate viral glycoprotein (GP)-mediated target cell binding [1–5]. Following attachment, FV capsids gain access to the host cell cytoplasm in a separate process upon GP-mediated fusion of viral and cellular lipid membranes that is HS-independent. For most FV species this process is preceded by endocytosis of bound virions, although direct plasma membrane fusion to a significant extent can be observed for prototype FV (PFV) [6–8]. Furthermore, FV GP-mediated membrane fusion appears to involve yet uncharacterized additional entry receptors essential for membrane fusion as HS-deficient target cell can still be infected in a FV GP-dependent manner, although at about 50- to 100-fold lower efficiency than respective HS-expressing cell types [1, 3–5].

**Fig. 1:**
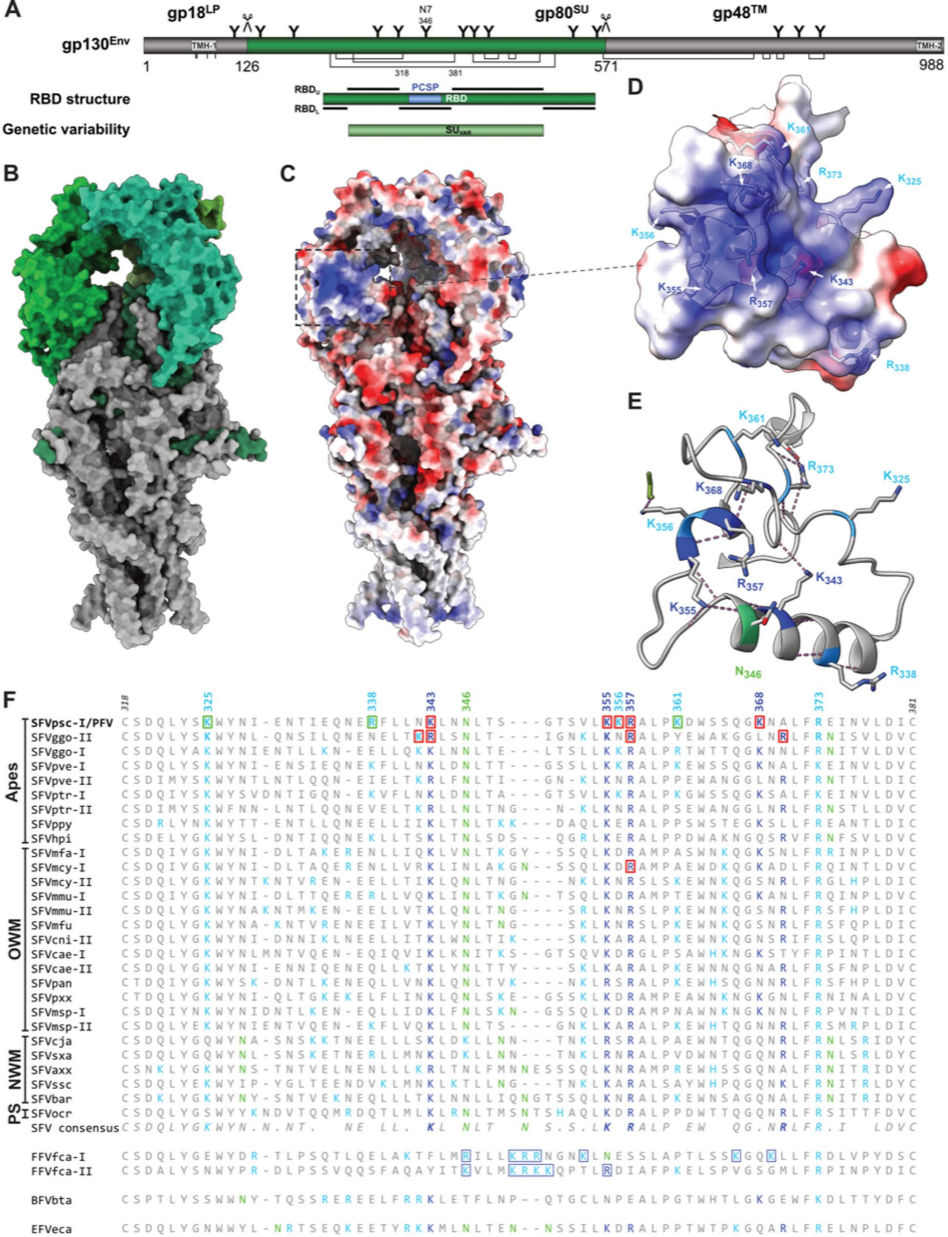
The PFV glycoprotein complex positively charged surface patch and putative heparan sulfate binding site. **A** Schematic illustration of the PFV Env precursor protein gp130 structural organization. The furin cleavage sites within the gp130^Env^ precursor used to generate the mature gp18^LP^, gp80^SU^, and gp48™ subunits are indicated by *scissor symbols*. The individual subunits are shown as *boxes* in different colors matching the structure in **B**. The positions of PFV Env N-glycosylation sites are marked by *Y-shaped symbols*. The positions of cysteine residues and disulfide bonds between individual cysteines are marked by *ticks* and *brackets* in *solid lines*, respectively. Hydrophobic sequences spanning the membrane in the gp18^LP^ (TMH-1) and the gp48™ (TMH-2) subunit are indicated as *grey boxes*. Below the SU region spanning the discontinuous, bipartite RBD is shown as a *box* with the segments forming RBD_L_ and RBD_U_ subdomains are indicated as *black bars*. The linear sequence of the positively charged surface patch (PCSP) is indicated as a light blue box. The SU_VAR_ region within the RBD differing between genotype I and -II Env proteins is indicated as a box. **B** 3D-structure cartoon of the PFV GPC trimer (PDB 8ozh) and **C** its electrostatic surface with **D**, **E** magnification of the C_318_ to C_381_ loop harboring the positively charged surface patch (PCSP). **D** The central (*dark blue*) as well as peripheral (*cyan*) positively charged residues and the N residue (*green*) of the non-essential N-glycosylation site 7 (22) are labeled as well as side chains shown, and **E** backbone- and side chain hydrogen bond interactions illustrated (*orchid*). The electrostatic potential in **C** and **D** colour palette goes from *red* (negative charge, −10 kcal mol^−1^ e^−1^) through *white* (neutral charge, 0 kcal mol^−1^ e^−1^) to *blue* (positive charge, +10 kcal mol^−1^ e^−1^). **F** Sequence alignment of the amino acid residues of the conserved GPC RBD subdomain of PFV (SFVpsc 318–381) that forms the PCSP harboring the putative heparan sulfate binding site of different SFV species of ape (Apes), old world monkey (OWM), new world monkey (NWM), or prosimian (PS) origin. If the Env genotype of individual SFV species are known they are indicated as appendix-I or -II to the species name shown on left. Central (*dark blue*) and peripheral (*cyan*) positively charged residues are highlighted in the individual FV species sequences and consensus sequences. The N residues of putative N-glycosylation site attachment consensus sequences are highlighted in *green*. Positively charged residues examined by mutagenesis in this study or by Fernandez et al. [13], which were found to be essential or non-essential for HS-dependent attachment and infectivity are highlighted by *red* and *green rectangles*, respectively. Non-primate FV Env residues with putative analogous function as the primate FV central PCSP residues are marked by *dark blue rectangles*

**Fig. 2:**
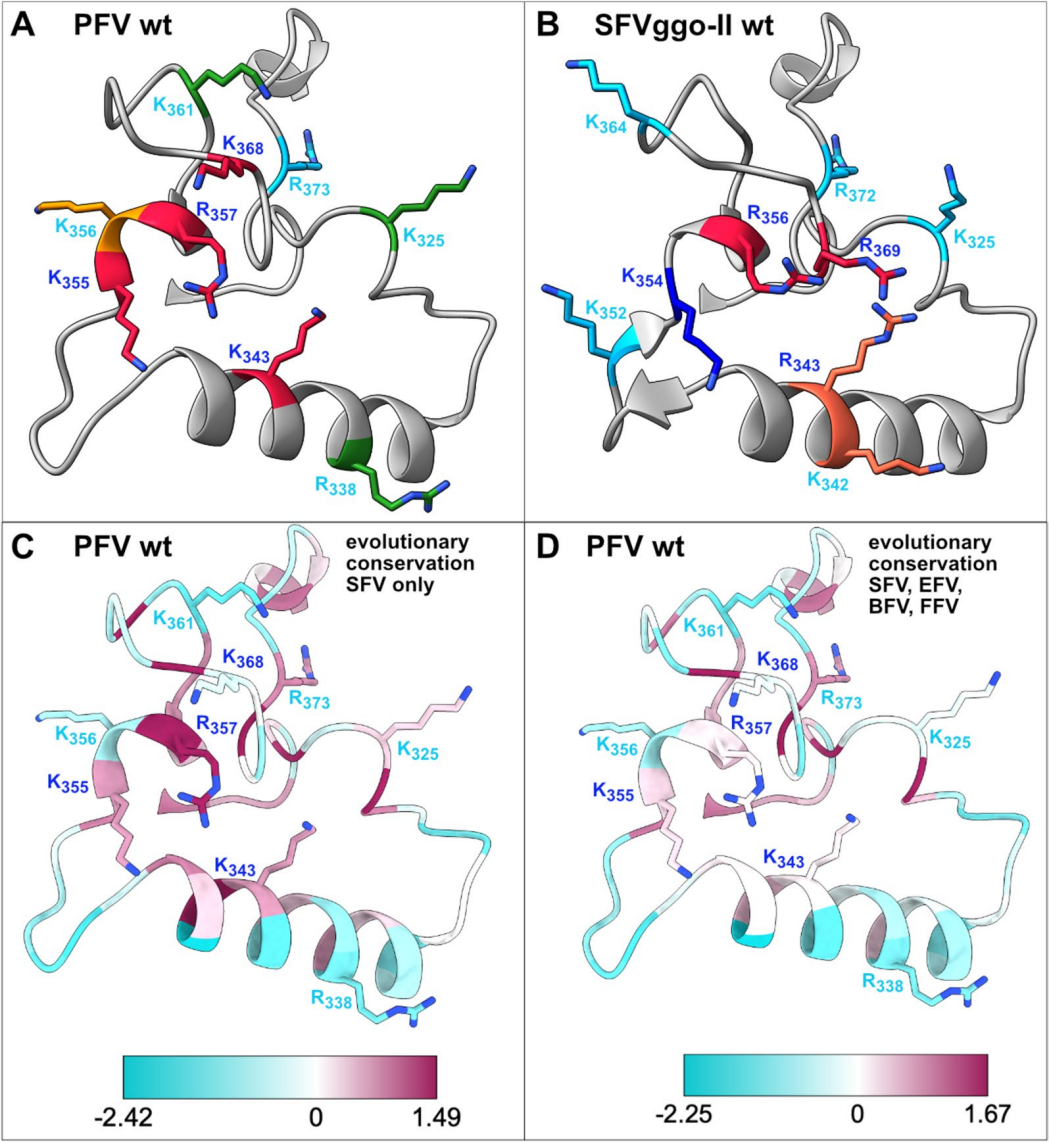
Comparison of PFV wildtype and SFVggo-II wildtype GPC PCSP loop structures. 3D-structure cartoon of **A**, **C**, **D** the wildtype PFV (PDB 8ozh) or **B** wildtype SFVggo-II (8aic) GPC of one protomers C318-C381/380 loop domain. **A**, **B** The side chains of the central (*blue labels*) and peripheral (*cyan labels*) positively charged residues of the PFV (**A**) and SFVggo-II (**B**) PCSP are shown. Residues (**A**) or residue pairs (**B**) with reported essential, contributing or dispensable functions for the GPC–HS-interaction as determined by this study (**A**) or by Fernandez et al. [13] (**B**) are colored in *red*, *orange* or *green*, respectively. **C**, **D** The evolutionary conservation of individual residues of the PFV GPC PCSP C318-C381 loop is indicated by coloring according to sequence conservation calculated using the AL2CO entropy-based measure [32], showing regions of low conservation (*cyan*) and high conservation (*maroon*). **C** Evolutionary conservation according to sequence alignment of various SFV species or **D** various SFV, FFV, BFV and EFV species listed in Fig. 1F

Mature FV glycoprotein complexes (GPCs) are organized as trimeric protrusions that are frequently arranged in elaborate lattice structures on the virion surface with hexamers and pentamers of Env trimers [9, 10]. Recently, high-resolution structures of GPC extracellular domains or membrane-embedded GPC from gorilla genotype II (SFVggo-II) and chimpanzee (SFVpsc or PFV) simian FV species became available [11–13]. They revealed a barrel-shaped FV GPC composed of three, intertwined protomers, each containing a leader peptide- (LP), a surface- (SU), and a transmembrane (TM) subunit, which are derived from an Env precursor by proteolytic processing during its passage through the cellular secretory pathway (Fig. 1A, B) [14, 15]. The bipartite receptor-binding-domain (RBD), located within SU, has a unique fold that forms a bean-like shape and is composed of two subdomains, RBD_U_ (upper RBD) and RBD_L_ (lower RBD) (Fig. 1A) [11, 13, 16]. Strikingly, two molecular FV *env* variants, termed genotype-I and -II, circulate in non-human primates (NHPs) and felines [17–21]. These Env variants differ in an approximately 250 residue long region within the GPC RBD, termed as variable SU (SU_VAR_) that is flanked by recombination hot spots (Fig. 1A). Phylogenetic analysis of SFVs suggest the ancient origin of the two Env variants dating to about 30 million years ago. The GPC structures of both SFVggo-II (genotype II) and PFV (genotype I) revealed positively charged surface patches (PCSP) at similar locations with RBD_L_ and the SU_VAR_ that represented putative HS binding sites (HSBS) (Fig. 1A, C, D; Fig. 2A, B) [11–13]. In the case of SFVggo-II GPC the patch’s function as HSBS was examined by mutagenesis, target cell binding, and infectivity studies. It revealed a functional role of two positively charged residue pairs (K_342_ + R_343_, R_356_ + R_369_) for HS binding (Fig. 2B) [13].

In this study we experimentally mapped the genotype I PFV GPC HSBS by functional analysis of eight positively charged residues in or in close proximity of the positively charged surface patch comprising its previously proposed HSBS within a disulfide-bond separated RBD subdomain (residue 318–381) (Fig. 1C) [11]. We generated PFV Env variants with point mutations, resulting in a switch of charge of individual positively charged residues, and characterized corresponding mutant vector particle assembly, infectivity and target cell binding characteristics. Thereby, we identified four PFV Env residues being individually absolutely essential (K_343_, K_355_, R_357_, K_368_), one residue with a consistent minor contribution (K_356_), and three residues (K_325_, R_338_, K_361_) not essentially required for the PFV GPC–HS interaction on different target cells (Fig. 2A).

## Results

### 3D-Structure based identification of positively charged residues forming the putative GPC HSBS

The recently determined structure of the membrane embedded GPC in PFV vector particles revealed a PCSP (Fig. 1B, C) at a similar location as reported for the SFVggo-II GPC ectodomain structure previously [11–13]. The PFV GPC PCSP appears to be composed of four positively charged, central residues K_343_, K_355_, R_357_, and K_368_ that are surrounded by five additional positively charged, peripheral residues, K_325_, R_338_, K_356_, K_361_, and R_373_ (Fig. 1C, D; Fig. 2A). Strikingly, the SFVggo-II GPC PCSP shows a similar organization of central (R_343_, K_354_, R_356_, R_369_) and peripheral (K_325_, K_342_, K_352_, K_364_, R_372_) positively charged residues (Fig. 2B). We decided to experimentally validate the potential function of eight solvent exposed residues for the PFV GPC–HS interaction on the surface of target cells by charge switch replacements (R/K to E). Peripheral residue R_373_ was not included in the analysis as it appears to be the least surface exposed, forms four hydrogen bond interactions with other GP residues (Fig. 1B–E), and is proposed to significantly contribute to the GPC structure. Therefore, its mutation has a high chance to induce strong alterations in the overall GPC 3D-structure resulting in a non-functional, misfolded protein. The other residues are more sovent exposed than R_373_ and display no or only up to two hydrogen bond interactions with neighbouring GP residues. The N-linked carbohydrate at residue N_346_ in this region (Fig. 1E, F) appears to be dispensable for cell binding and infectivity [22]. We generated a set of packaging constructs encoding PFV Env variants with R/K to E exchanges of these residues.

### Surface patch central, positive residues are essential for HS-dependent enhancement of FV GPC-mediated vector infectivity

**Fig. 3:**
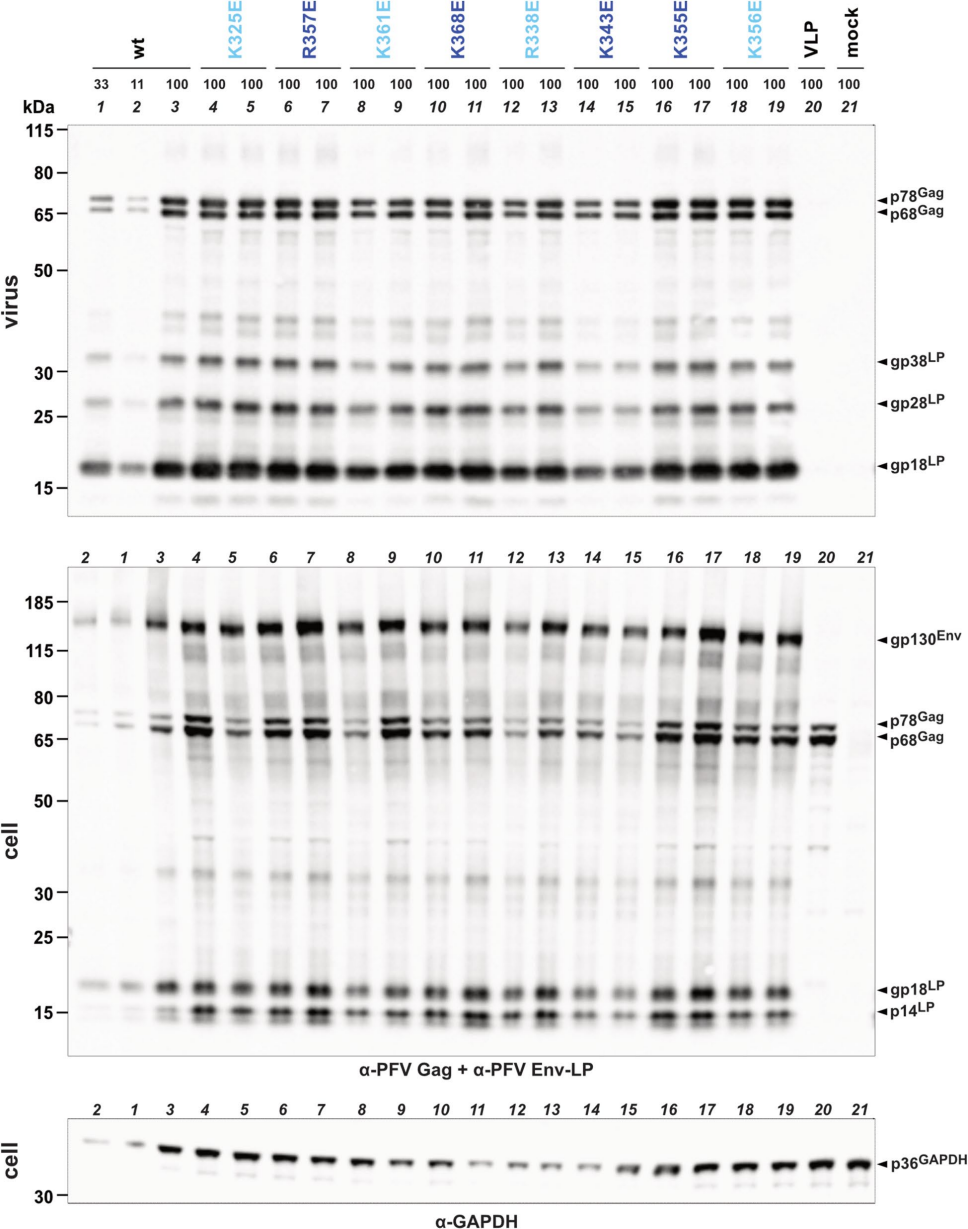
Analysis of cellular expression and physical particle release. Cell-free viral supernatants harboring different GPs as indicated, or control supernatants containing no GP (virus like particle, VLP), or coming from a mock transfection with pUC19 (mock), were generated by transient transfection using a 4-component PFV vector system and virus particles concentrated by ultracentrifugation. Physical particle release and cellular expression was determined by Western blot using equal volumes (100% sample) of concentrated viral particle lysates (virus) and cell lysates (cell) of the individual samples, respectively, separated by SDS-PAGE and immunoblotting using PFV Gag and PFV Env LP subunit- or GAPDH-specific polyclonal antisera. A threefold serial dilution of the wildtype (wt) sample is shown in lane 1 to 3. Shown are the results of a representative experiment (n = 3) with two independent clones for each mutant PFV Env packaging construct variant

Replication-deficient, EGFP expressing PFV vector particles harboring parental wildtype or the individual PCSP GPC variants, as indicated, were generated by transient transfection of 293T-25A packaging cells. Biochemical analysis of cellular viral protein expression, physical particle release and protein composition as well as genetic analysis of particle-associated viral nucleic acid composition revealed no major differences of all PFV Env mutants compared to the parental wildtype control (Fig. 3; Fig. S1).

**Fig. 4:**
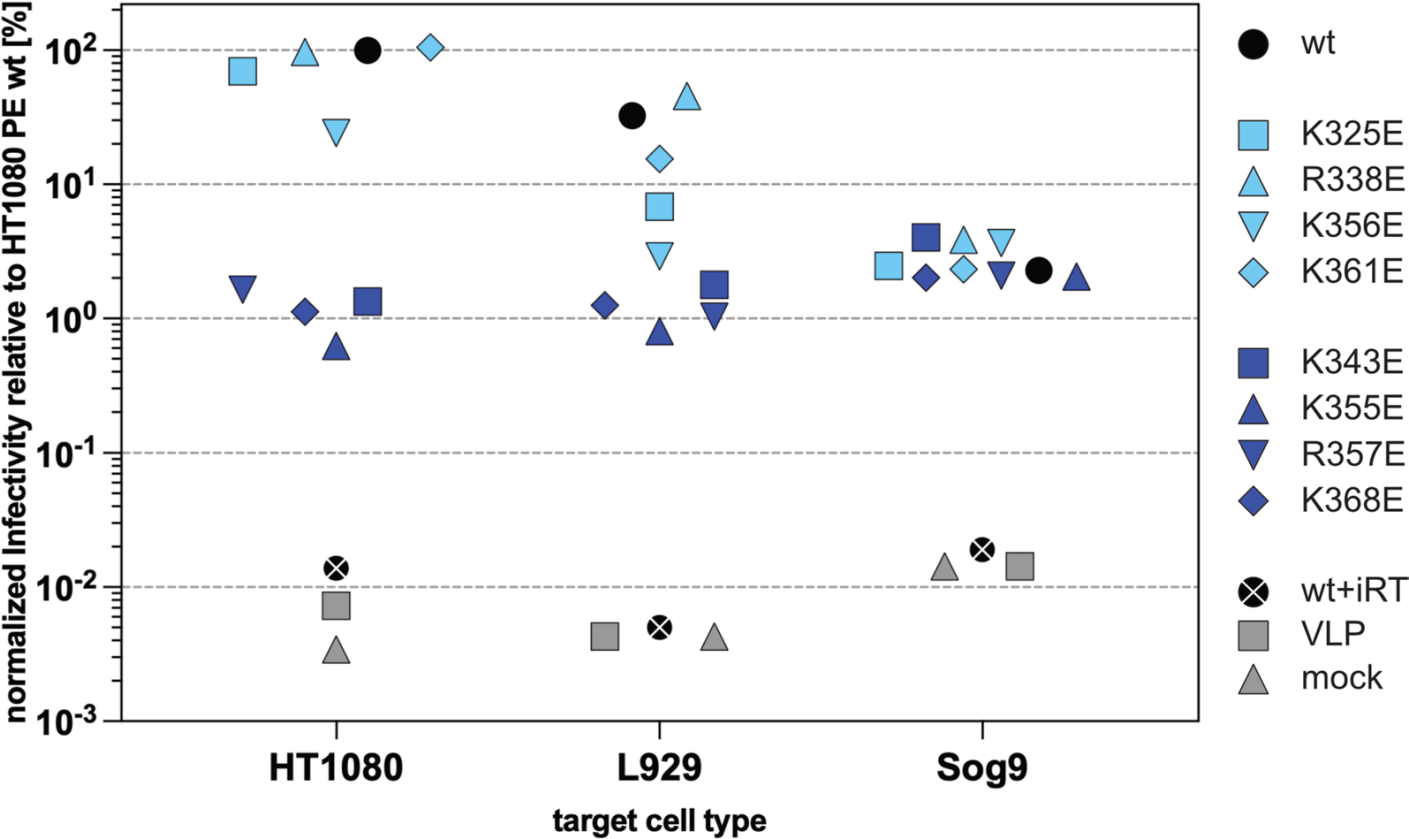
Infectivity of various PFV GPC mutant containing vector supernatants on different target cells. Cell-free viral supernatants, generated by transient transfection using a 4-component PFV vector system, and harboring different GPs as indicated (peripheral PCSP mutants *light blue symbols*; central PCSP mutants *dark blue symbols*), or control supernatants harboring wt GP (wt, *black circle*) and enzymatically inactive RT (wt + iRT, *black circle* + *white cross*), containing no GP (no GP, *grey square*), or coming from a mock transfection with pUC19 (mock, *grey triangle*), were titrated on different target cells as indicated. Viral titers were calculated from the flow cytometric determination of GFP expression 72 h.p.i.. vDNA content was determined by qPCR from pelleted viral particle nucleic acid extract. Shown are Mean (n = 5–8) of relative infectivity compared to wt PFV GP (wt) containing supernatants on HT1080 cells. Except for the ctrls (wt + iRT, VLP, mock) relative infectivity values were normalized for physical particle (vDNA) content in comparison to the wt PFV GP (wt) sample on HT1080 target cells

Subsequently, the vector supernatants were titrated on target cells of different species and varying in their HS-cell surface expression level [1] to evaluate the functional role of individual residue alterations for viral infectivity. Titration analysis on HS-expressing HT1080 cells allowed distinction of three groups of mutants in respect to their infectivity phenotypes (Fig. 4; Fig. S2). The first group includes PFV Env peripheral PCSP mutants K_325_E, R_338_E, and K_361_E, whose vector supernatants displayed a wt-like infectivity. The second group, the only member being peripheral PCSP mutant K_356_E, had a fourfold reduced infectivity. The infectivity of members within the third group, composed of central PCSP mutants K_343_E, K_355_E, R_357_E, and K_368_E, was 50- to 200-fold lower compared to wildtype. In general most of the individual GP mutant containing supernatants displayed a similar infectivity pattern on HS-expressing mouse L929 target cells (Fig. 4; Fig. S2). Though, unlike to HT1080 target cells, the infectivity of group 1 GP mutants K_325_E and R_361_E containing supernatants was significantly (threefold to sevenfold) reduced compared to the wild type control whereas only that of R_338_E GP containing supernatants remained wild type-like. Thus K_325_E and R_361_E would be characterized as group 2 mutants on mouse L929 cells. Furthermore, the infectivity reduction of the group 2 peripheral GP mutant K_356_E supernatant was more pronounced on mouse L929 cells (20-fold) compared to HT1080 (sevenfold).

As reported previously [1], vector supernatant infectivity of wildtype PFV GPC containing particles was reduced 10- to 30-fold on HS-deficient mouse Sog9 cells compared to parental mouse L929 cell controls (Fig. 4; Fig. S2). In contrast to titration on HT1080 or mouse L929 cells, vector supernatants containing wildtype PFV GPC or the individual PCSP mutant GPCs showed similar infectivities on Sog9 target cells. However, a residual, basal infectivity, of about 100-fold above various negative controls, was still detectable for wildtype and all mutant PFV GPC containing vector particles. This confirmed previous findings [1, 3–5] that HS is an attachment but not an essential membrane fusion receptor of PFV Env-mediated target cell entry. Together the mouse L929 and Sog9 cell data suggest that HS expression, and the failure of individual mutant GPCs to interact with it, is responsible for the differential infectivity phenotypes observed for the PFV GPC variants examined.

In summary, the four positively charged, central residues K_343_, K_355_, R_357_, and K_368_ of the PFV GPC PCSP appear to functionally constitute its major HSBS. Of the peripheral residues only K_356_ seems to consistently contribute to some extent to HS-dependent infectivity enhancement, though at lower level than the four positively charged, central residues.

### HS-dependent cell surface binding of wildtype and mutant virions

Next, we examined binding of PFV virions bearing different GPC variants to various target cells using two alternative assays. The first assay is based on flow cytometric detection of GFP tagged capsids bound to target cells and was applied in previous studies from our laboratory [5, 23]. Strikingly, the analysis of the different PFV particles on H1080 revealed that for three PFV GPC variants, their binding capacity did not correlate well with their infectivity phenotype determined using vector supernatants without GFP-tagged Gag (Fig. 4; Fig. S2; Fig. S3). PFV vector particles containing K_325_E, K_356_E, or K_361_E mutant GPCs, that displayed infectivities of 69%, 24%, or 105% relative to wildtype on HT1080 cells (Fig. S3C), achieved binding capacities of only about 14%, 4% and 18% of wildtype (Fig. S3B), respectively. In contrast, R_338_E GPC containing particles showed 72% wildtype binding capacity that correlated much better with their relative infectivity of 97% of wildtype (Fig. S3B, C). For all other mutant GPC (K_343_E, K_355_E, R_357_E, K_368_E) bearing particles no or only very little specific target cell binding was detectable, corresponding to levels below 4% of wild type and close to the detection limit of ~1% (Fig. S3B, C). A similar relative binding profile was observed for the respective GPC mutant containing PFV particles on HS-expressing mouse L929 cells (Fig. S3B). Strikingly, the infectivity and binding profile of all individual mutant GPC particles, including K_325_E, K_356_E, and K_361_E, correlated much better on mouse L929 cells than on HT1080 cells (Fig. S3B, C). On HS-deficient Sog9 cells all GPC mutant containing particles showed a similar, wild type-like, binding capacity, that was clearly above the detection limit of the assay (Fig. S3B, C).

**Fig. 5:**
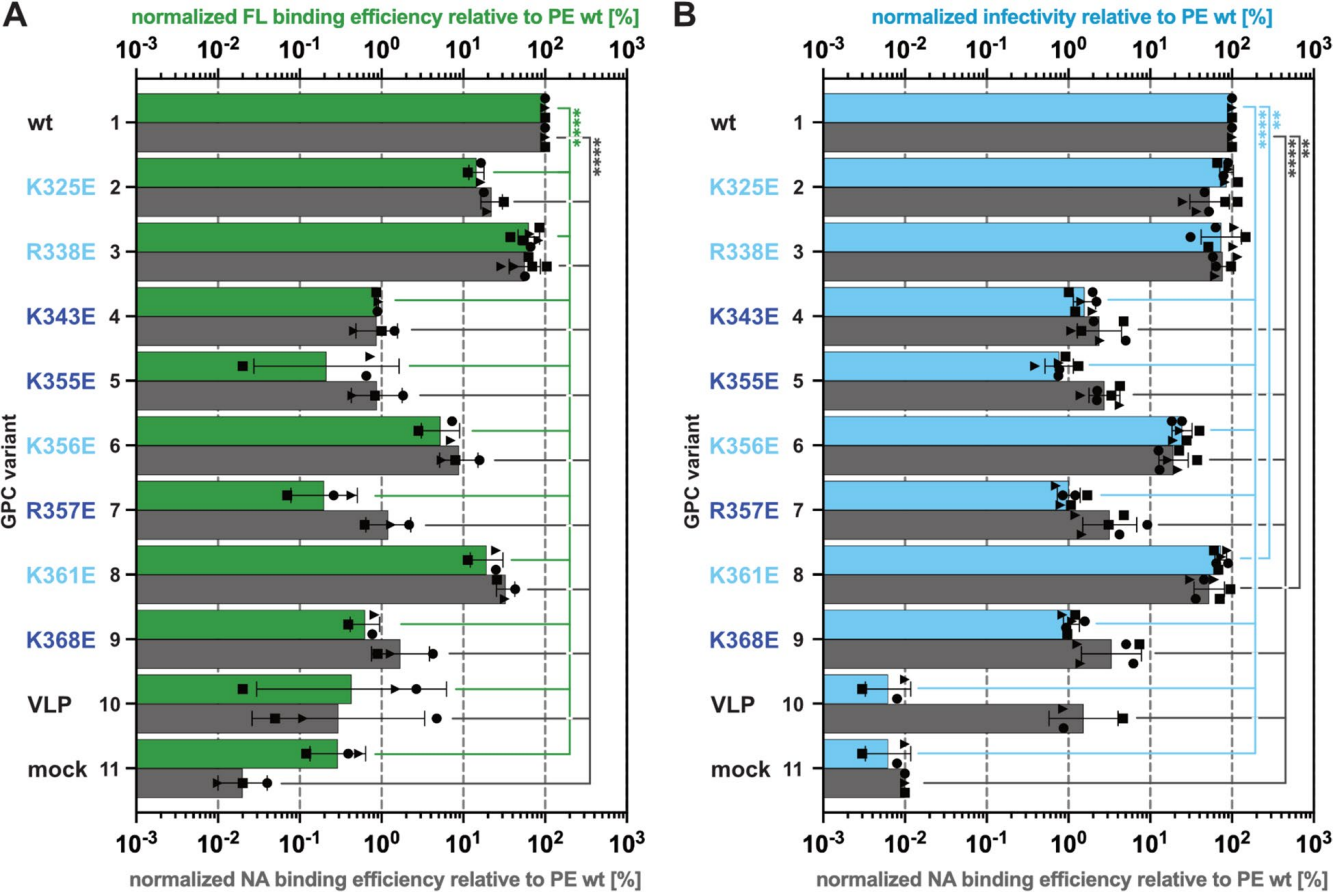
Comparison of different virus binding assays. Plain, cell-free viral supernatants, generated by transient transfection using a 4-component PFV vector system, and harboring GFP-tagged (**A**) or untagged (**B**) PFV Gag in combination with different GPs as indicated, or control supernatants containing no GP (VLP) or no viral proteins (mock) were incubated with different target cells and analyzed by flow cytometry as described in material and methods. **A** Summary of relative binding efficiencies of PFV GPC containing or control vector supernatants on HT1080 using the fluorescent (FL, *dark green bars*) or nucleic acid (NA, *dark grey bars*) cell binding assay. Shown are Mean ± SD (n = 3–6) of three independent vector supernatant productions. **B** Summary of relative infection (*light blue bars*) and binding efficiencies (NA, *dark grey bars*) of individual PFV GPC containing or control vector supernatants on HT1080 cells. Shown are Mean ± SD (n = 3–6) of three independent vector supernatant productions. *ns* non-significant, *p < 0.05, **p < 0.01, ***p < 0.001, ****p < 0.0001, by ordinary two-way ANOVA followed by Tukey’s multiple comparison test

Fernandez and colleagues identified four positively charged residues of the SFVggo-II GPC as representing the FV species HSBS (Fig. 2B) [13]. In their study a qPCR based binding assay quantifying particle-associated viral nucleic acids was employed. To determine whether the poor correlation of HT1080 target cell binding and infectivity of the PFV GPC K_325_E, K_356_E, and K_361_E containing virions is dependent on the fluorescent binding assay employed we established a second qPCR-based assay similar to the one of Fernandez and colleagues [13]. HT1080 target cells were then incubated at low temperature and spinoculation with vRNA copy number normalized supernatants of Gag-GFP containing PFV particles harboring the individual GPC variants in duplicates to directly compare the results of the different readouts, mean fluorescence intensity- vs. vRNA copy number quantification, using the same virus preparations. The results of both assays revealed a similar HT1080 cell-binding profile for the individual GPC variant containing PFV supernatants (Fig. 5A). Again K_325_E, K_356_E and K_361_E GPC containing particles showed a reduced cell-binding capacity that did not match their HT1080 infectivity phenotype (Fig. 5A, Fig. S3C). Thus the HT1080 cell binding phenotype of the individual GPC variant containing supernatants was not dependent on the readout of the binding assay type employed.

Previous studies [5] revealed that PFV Gag-GFP tagged virions, similar to those examined in the different cell binding assays described above, have a strongly reduced infectivity as a consequence of a diminished reverse transcription (RTr). Therefore, the infectivity and cell binding characteristics of GPC variant containing virions described above were obtained from different types and preparations of PFV particles and may have been the reason for the observed discrepancy in HT1080 binding and infectivity characteristics of the K_325_E, K_356_E, and K_361_E GPC mutant particles. To better compare and correlate infectivity and cell-binding characteristics the qPCR-based binding assay was employed for fully infectious PFV vector supernatants containing untagged Gag. HT1080 cells were incubated in duplicates, at low temperature and spinoculation, with vRNA copy number normalized vector supernatants harboring the individual GPC variants. After removing unbound PFV particles one duplicate sample was immediately subjected to qPCR-based quantification of cell-associated vRNA and ß-actin gene copy number determination. The other sample was further cultivated and infectivity determined by flow cytometric quantification of GFP reporter gene expressing target cell frequencies 72 h later. The analysis of physical particle normalized virus infectivity and binding to HT1080 using the same virus supernatants generally showed a similar attachment phenotype for the individual GPC PCSP mutants as observed with the fluorescent binding assay (Fig. 5B; grey bars). Again, all central PCSP GPC mutants (K_343_E, K_355_E, R_357_E, and K_368_E) displayed the lowest target cell binding potential at levels of 2–5% of the wild type control and only slightly higher than VLPs without GPC of 1.5% (Fig. 5B; grey bar 1, 4, 5, 7, 9, 12). Peripheral GPC PCSP mutants K_325_E, R_338_E and K_361_ showed binding efficiencies of at least 50% of wild type whereas that of K_356_E was reduced down to 20% (Fig. 5B; grey bar 1, 2, 3, 6, 8). However, the binding efficiency of all peripheral PCSP mutants to HT1080 cells now correlated much better with their respective infectivities (Fig. 5B; compare light blue and grey bar 2, 3, 6, 8). Thus the GFP-tag on the PFV capsids appears to influence the HT1080 target cell binding characteristics of some peripheral PCSP residue variants that retain residual interaction potential with HS.

## Discussion

In this study we functionally assessed the PFV GPC HSBS. We show that positively charged residues within a PCSP, that was first observed on the recently solved structures of PFV [11] and SFVggo-II GPC [13], are the key determinants for HS-dependent attachment to target cells. The organization of positively charged residues within PFV and SFVggo-II GPC PCSPs, located in disulfide-bond separated GPC RBD subdomains is strikingly similar. They are organized in four central and five peripheral positions within the PCSP (Fig. 1C–E; Fig. 2A, B). Comparing the essential positively charged residues for PFV HSBSs functionally identified in this study to those of SFVggo-II characterized by Fernandez and colleagues [13] reveals a conservation of the two GPCs HSBS. Unfortunately, for SFVggo-II GPC only double residue mutants (K_342_ + R_343_, R_356_ + R_369_) were examined and not single residue substitutions as in this study. Therefore, based on our results obtained for PFV GPC, we can only speculate about the importance of individual, positively charged SFVggo-II residues for its interaction with HS. Three of the essential PFV GPC central residues, K_343_, K_355_, and R_357_, have analogous essential residues, R_343_, K_354_, and R_356_, at the same location in the SFVggo-II GPC (Fig. 2A, B). Strikingly, they are evolutionarily conserved in all primate FV species (Fig. 1F ; Fig. 2C). Alteration of SFVggo-II GPC R_343_ or R_356_, each in combination with with a second residue. was shown to abolish the HS-interaction whereas K_354_ was not examined [13]. The fourth essential central PFV GPC residue K_368_ has no perfect, evolutionarily conserved counterpart in SFVggo-II GPC (Fig. 1F). However, SFVggo-II GPC R_369_ is localized in close proximity to PFV GPC K_368_ and was shown, in combination with R_356_, to be essential for the HS-interaction as well (Fig. 2A, B). Similarly, SFVggo-II GPC residue K_342_ examined by Fernandez and colleagues [13] in combination with R_343_, has no perfect counterpart in the PFV GPC. However, PFV GPC peripheral residue R_338_ is localized in close proximity and was found to be dispensable for the GPC–HS interaction. Therefore we postulate that SFVggo-II GPC K_342_ alone is also dispensable for the GPC’s HS interaction.

Taken together our analysis of single PFV GPC residues clearly shows that the four central residues (PFV GPC K_343_, K_355_, R_357_, and K_368_) are the most essential determinants as individual charge switch residues replacements reduced HS-dependent target cell attachment to baseline levels. The functional conservation of this sequence motif amongst primate FV species is further supported by the demonstration that a R_358_E charge switch mutant of SFVmcy-I GPC (analog of PFV GPC R_357_E), also strongly reduces HS-dependent infectivity (Fig. S4). Sequence alignment of the Env RBD loop of various primate FV species corresponding the PFV Env residues 318 to 381 reveals that all contain 8 to 11 positively charged residues (Fig. 1F). The location of the first three central, positively charged residues analogous to PFV Env position 343, 355 and 357 is evolutionarily conserved amongst all primate FV species regardless of their Env genotype (Fig. 1F; Fig. 2C). The fourth central positively charged residue, found in all SFV Envs except SFVaxx and SFVbar, is located either at PFV GPC analogous position 368 or 370 (Fig. 1F). Strikingly, for all SFV species with characterized isolates of different Env genotypes the fourth central positively charged residue is found at position 368 in the genotype I variant whereas it is located at position 370 in the genotype II variant.

So far no Env genotype variants have been described for NWM SFV species. The Env proteins of SFVcja, SFVsxa and SFVssc isolates contain a positively charged central residue at PFV corresponding position 370 whereas those of SFVaxx and SFVbar lack a fourth central, positively charged residue (Fig. 1F). Assuming that also NWM harbor yet unidentified SFV variants with different Env genotypes we hypothesize that the currently known SFVcja, SFVsxa and SFVssc isolates represent genotype II variants whereas the SFVaxx and SFVbar isolates are of genotype I origin. The latter may therefore deviate from genotype I variants of OWM and Apes by lacking a fourth central positively charged HSBS residue.

In contrast to the central positively charged residues of the PFV GPC PCSP, of the four peripheral residues examined only K_356_ appears to significantly contribute in a consistent manner to HS-dependent attachment on the examined HS-expressing human and mouse cell lines. Though at a much lower level than the central residues. Strikingly, K_356_ is only found in the Ape genotype I variant GPCs of PFV/SFVpsc, SFVggo, SFVpve, and SFVptr isolates and absent from all other primate FV GPCs (Fig. 1F). Therefore we postulate also for the other primate FV GPCs only a minor contribution of peripheral positively charged residues of the PCSP to HS-interaction. However, since we observed differences in the level of the peripheral residue’s contribution to HS-dependent attachment depending on the target cell type examined, HT1080 vs mouse L929, this may not be true for all target cell types. Perhaps different HS-compositions of the target cells are responsible for this phenomenon, a factor that should be examined in future studies using single residue replacement mutants of FV GPCs for the two Env genotype variants.

Among the positively charged peripheral residues of the PFV GPC, only K_325_ and R_373_ appear to be evolutionarily conserved within primates and between primate and non-primate FV species, respectively (Fig. 1F; Fig. 2C, D). No other peripheral residues show evolutionary conservation, even among primate FV species alone. The conservation of the buried residue R_373_ appears to be due to structural constraints, whereas the conservation of the surface-exposed residue K_325_ may stem from a potential cell-type-specific contribution to the GPC–HS interaction. As illustrated by the data in Fig. 4 and Fig. S2, the infectivity of the K_325_E GPC mutant shows the second-largest reduction among all peripheral residue mutants in mouse L929 cells. Determining the infectivity-binding phenotype of the PFV Env GPC K_325_E mutant across additional target cell types—perhaps varying in their HS composition—may further clarify whether this interaction is the functional basis for the residue’s evolutionary conservation.

FFV Env is the only non-primate FV GPC with experimentally verified HS-dependent infectivity [3]. Similar to PFV Env, FFV Env-dependent infectivity was inhibited by heparin in a dose-dependent manner with comparable IC_50_ values. Strikingly, sequence alignment of primate and non-primate FV Env species reveals that genotype I FFV Env lacks the four central PCSP residues typically conserved in primate FV Envs (Fig. 1F). Genotype II FFV Env retains only one of these four residues (analogous to PFV Env position 355); in genotype I, this same position is occupied by an asparagine within an N-glycosylation site consensus sequence. Interestingly, both FFV Env genotypes contain a cluster of three or four positively charged residues inserted between the positions analogous to PFV Env residue 349/350. These may serve as functional substitutes for the missing primate central PCSP residues, though this remains to be tested experimentally.

In contrast to FFV, the Env proteins of BFV and EFV possess either two (the first and fourth) or all four central PCSP residues at the evolutionarily conserved positions identified in primate FV Envs (Fig. 1F). Furthermore, both Env proteins contain two positively charged residues preceding the first central PCSP residue (analogous to the corresponding PFV Env residue K_343_), which may contribute to the HS-dependent attachment of these FV species.

Although multiple BFV and EFV Env sequences are available in GenBank, no genotype-specific Env variants have been identified to date. However, if the "rule" regarding the genotype-specific location of the fourth central PCSP residue in primate FV is applied to BFV and EFV Envs, all known BFV Env isolates would be classified as genotype I, while all EFV isolates would be genotype II.

Taken together the functional data of this study for PFV and SFVmcy-I Env as well as the results of Fernandez and colleagues for SFVggo-II, in combination with bioinformatic analysis of the Env PCSP domain sequence of different FV species, strongly suggest that all primate FV as well as BFV and EFV GPC contain an evolutionarily conserved HSBS composed of two to four central positively charged residues located in a PCSP of the GPC RBD.

## Conclusion

In summary, the results of our study functionally characterizing the PFV GPC HSBS strongly support the notion first voiced by Fernandez et al. [13] that an evolutionarily conserved PCSP, observed in the recently solved structures of SFVggo-II and PFV GPCs, harbors the primary determinants for HS-dependent attachment to an infectivity enhancement of target cells. Centrally located positively charged residues within the PCSP of primate FVs and potentially some non-primate FV species are essential determinants for this process. These central residues constitute the GPCs major HSBS whereas additional peripherally located ones are not or only weakly involved. Charge-switch alterations of single, essential, positively charged residues are sufficient to completely neutralize HS-dependent target cell attachment of FV particles and strongly reduce their specific infectivity.

## Methods

### Cells and culture conditions

The human epithelial fibrosarcoma cell line HT1080 (ATCC CCL-121, [24]), the human embryonic kidney cell line 293T (ATCC CRL-1573, [25]) as well as its HS-deficient variant 293T-25A [1], and mouse fibroblast L929 cells (ATCC CRL-264, [26]) as well as its HS-deficient variant Sog9 (F. Neipel, Erlangen) were cultivated at 37 °C, 5% CO_2_ and 95% humidity in complete Dulbecco’s modified Eagle’s medium (DMEM) supplemented with 10% heat-inactivated fetal calf serum and antibiotics.

### Recombinant DNAs and plasmid expression constructs

A four-component vector system was used to generate integration-competent but replication-deficient, single-round PFV vector supernatants. It consists of the transfer vector plasmid puc2MD9 (containing an U3 (SFFV U3) promotor-driven EGFP reporter gene expression cassette) and the expression-optimized packaging plasmids pcoPG4 (PFV Gag), pcoPP (PFV Pol), pcoPE (PFV Env), pcoSE (SFVmcy-I Env) as described previously [5, 27]. For production of fluorescent protein tagged PFV particles the transfer vector plasmid puc2MD9 Ubi DsRedEx2, containing a ubiquitin C promotor-driven DsRed Express 2 reporter gene expression cassette, and pcoPG 1-621 CEG [23], expressing a C-terminal tagged PFV p68^Gag^ protein, were used instead of puc2MD9 and pcoPG4. For this study, various glycoprotein packaging constructs encoding different PFV Env variants with single or multiple specific amino acid replacements were generated by recombinant PCR techniques: pcoPE-∆HS1a (K325E), -∆HS1b (R357E), -∆HS1c (K361E), -∆HS1d (K368E), -∆HS1e (R338E), -∆HS1f (K343E), -∆HS1g (K355E) and -∆HS1h (K356E). Furthermore, a SFVmcy-I glycoprotein variant pcoSE-∆HS1b (R358E) was generated. All constructs were verified by Sanger sequencing analysis. Additional details as well as sequences of primers are available upon request.

For qPCR analysis the following reference plasmids containing the individual primer–probe set-specific target sequence were used to prepare copy number standards: pCR.2.1-TOPO-ACTB (human ß-actin); pk2HSRV2 1-LTR-Circle (PFV vgRNA or vgDNA).

### Viral supernatant production and concentration

FV vector particles were produced as described previously [1]. Briefly, the individual plasmids of the 4-component PFV vector system were co-transfected transiently into 293T-25A packaging cells using calcium phosphate (CaP) co-precipitation in 10 cm dishes. 293T-25A cells were co-transfected with different combinations of PFV Gag and glycoprotein expression constructs as indicated. In experiments examining the infectivity of viral supernatants the PFV or SFVmcy-I Env expression constructs were co-transfected together with PFV Pol (pcoPP) and PFV Gag (pcoPG4) packaging constructs, as well as the transfer vector (puc2MD9) at the ratio of 1:1.5:3.25:13 to an amount of 30 µg total transfected DNA per dish. For production of fluorescent protein tagged PFV particles puc2MD9 and pcoPG4 were replaced by puc2MD9 Ubi DsRedEx2 and pcoPG 1-621 CEG expressing a C-terminal EGFP-tagged PFV p68^Gag^ protein, respectively. Cell-free viral vector supernatant was harvested 72 h post transfection using 0.45 μm sterile filters (10 cm dish) and stored in aliquots at −80 °C until further use in cell attachment or infectivity assays as described below.

For analysis of particle protein and nucleic acid composition supernatants were concentrated (100× volume-fold) by ultracentrifugation essentially as described previously [28, 29]. Twelve or thirty milliliter plain, cell-free vector supernatant samples were pelleted by centrifugation in SW-32Ti rotors at 25,000 rpm (~82,600 g_ave_), at 4 °C for 90 min. The supernatant was discarded and the viral pellet was gently resuspended in 120 µl PBS containing 3.4 U/µl DNase I and incubated for 1 h at 37 °C. Subsequently DNase I digested particle samples were stored at −80 °C until further nucleic acid extraction or generation of protein lysates.

### Analysis of viral infectivity

Infectivity of viral supernatants produced was determined by a flow cytometric based EGFP marker gene transfer assay similar as described previously [5]. Target cells were seeded at the density of 1 × 10^4^ cells/well in 96-well plates 16–24 h prior to transduction. Target cells were incubated for 4–6 h with 100 µl of cell free viral supernatant, generated by transient transfection as described above, and tenfold serial dilutions thereof before replacement with normal growth medium and further incubation at 37 °C and 5% CO_2_. The percentage of EGFP marker gene expressing cell was determined 72 h post infection (h.p.i.) by flow cytometry using a MACSQuant VYB flow cytometer (Miltenyi Biotech). All transduction experiments were performed at least three times and, in each independent experiment, the titers obtained with the untagged wild-type viruses were arbitrarily set to 100% and those of the other samples expressed as values relative to the wt control, as described previously [30].

### Analysis of particle cell attachment

For analysis of PFV particle binding to target cells two different assays were applied.

#### Flow cytometry assay

The flow cytometry-based particle attachment assay was performed as described previously [23]. Briefly, 1 × 10^5^ cells/well in 12-well plates plated 16–24 h prior to analysis were incubated at 10 °C with either 500 µL cold, plain undiluted GFP-tagged p68^Gag^ protein containing vector supernatant harboring the individual GPC variants, or alternatively using physical particle normalized amounts of plain vector supernatant corresponding to 1 × 10^6^ vgRNA copies (MOI 10) in a total volume of 500 µl, and spinoculated at 980×*g*, 10 °C for 45 min to facility particle cell surface binding and prevent endocytic uptake. For PFV Env wt containing particles a threefold serial dilution of vector supernatants was used to allow generation of a reference standard. Subsequently, the vector supernatant was aspirated, the cells were washed once with cold PBS and lifted of by trypsination at 4 °C. Finally, the mean (GFP) fluorescence intensity (MFI) of the whole cell population of individual samples and appropriate controls was determined by flow cytometry and FlowJo analysis. Titration of wild type PFV Env containing vector supernatant demonstrated that with the assay up to 81-fold reduced binding was detectable on HT1080 target cells (Fig. S3A).

#### qPCR assay

For the qPCR-based particle attachment assay target cells were plated and incubated with cell-free viral vector supernatants contain capsids with or without GFP-tag as described for the flow cytometry assay. After aspiration of the viral supernatant cell layers were washed twice with cold PBS prior to addition of the lysis buffer of the RNeasy Mini Kit (Qiagen). Subsequently nucleic acids were extracted following the manufacturers’ instructions and eluted in a total volume of 50 µL. For RT-qPCR analysis of vgRNA content 15 µl nucleic acid (20 ng/µl) samples were DNase I digested using the RapidOut DNA Removal Kit (ThermoFisher Scientific) in a total volume of 20 µl. Five microliter thereof, or of a tenfold dilution series of a plasmid copy number standard (corresponding to 10^8^ to 10^2^ copies/5 µl), were subsequently used in RT-qPCR assays using the Luna Universal Probe One-Step RT qPCR Kit (New England Biolabs) in combination with a PFV U5-Psi primer–probe set (Table S1). For ß-actin gene copy number determination 5 µl of a 20 ng/µl dilution of the extracted nucleic acid samples, or of a tenfold dilution series of a plasmid copy number standard, were used in qPCR assays using the Luna Universal Probe qPCR Master mix in combination with a human ß-actin primer–probe set (Table S1). RT-qPCR and qPCR assays were run on a CFX Opus 96 (BioRad) Realtime PCR Thermal Cycler and analyzed using the CFX Maestro™ software package.

### Quantitative PCR analysis

Following viral particle concentration and DNase I digest of intact particles as described above in “Viral supernatant production and concentration” viral particle-associated nucleic acids were extracted using the QIAmp Viral RNA Mini Kit (Qiagen) according to the manufacturers’ instructions without the optional DNAse I digest and eluted in 60 µl. RT-qPCR analysis of vgRNA and qPCR analysis of vgDNA content were performed as described above in “Analysis of particle cell attachment, qPCR assay” using primer–probe sets summarized in Table S1.

### Western blot analysis and antisera

Cellular lysates for protein expression analysis were prepared by washing the transfected 293T-25A cells in 10 cm dishes with PBS and following 20 min of incubation with 600 µl cell-lysis buffer (10 mM Tris/HCl pH 8.0, 140 mM NaCl, 0.025% NaN_3_, 1% Triton-100) on ice. Subsequently, cell lysates were scraped off the cell culture dishes and centrifuged through a QIAshredder (QIAGEN). After rinsing the QIAshredder with 600 µl 2 × PPPC (100 mM Tris–HCl; pH 6.8, 24% Glycerol, 8% SDS, 0.02% Coomassie Brilliant Blue G-250, 2% ß-Mercaptoethanol) the combined fractions of flow through were boiled at 95 °C for 10 min and used directly for SDS–polyacrylamide gel electrophoresis or stored at −20 °C until further use.

For analysis of viral particle-associated protein composition cell-free supernatant of transfected 293T-25A cells (10 cm dish) was harvested using a syringe and a 0.45 µm pore size sterile filter. Viral particles were concentrated by ultracentrifugation at 25,000 rpm, 4 °C in SW32 (76,755×*g*) or SW40 (78,925 × *g*) rotors through a 20% sucrose cushion. Viral pellets were resuspended in 50 µl PBS and 50 µl 2 × PPPC was added. After boiling for 10 min at 95 °C the samples were used directly for SDS–polyacrylamide gel electrophoresis (SDS-PAGE) or stored at −20 °C.

Cell or viral particle protein samples were separated by SDS-PAGE using a 7.5% polyacrylamide gel and analyzed by immunoblotting as described previously [31]. Polyclonal rabbit antisera specific for PFV Gag [29] or for the PFV Env LP subunit [31] were used. After incubation with a suitable horseradish peroxidase (HRP)-conjugated secondary antibody, the blots were developed with Immobilon Western HRP substrate. The chemiluminescence signal was digitally recorded using an iBright FL1500 imager (Thermo Fisher Scientific).

### Bioinformatic sequence analysis

Sequences alignments of FV Env proteins were performed using the multiple sequence alignment function of the MacVector (v18.8.2) software package employing the ClustalW algorithm. The Env sequences used in the alignment shown in Fig. 1F were obtained from public databases and with following accession numbers: SFVpsc-I/PFV huHSRV13 (known as Prototype Foamy Virus genotype I Eastern chimpanzee SFV; Genbank AQM52259.1); SFVggo-I huBAD468 (genotype I gorilla SFV; GenBank: AFX98095.1); SFVggo-II huBAK74 (genotype II gorilla SFV, Genbank AFX98090.1); SFVpve-I SFVcpz (genotype I Western chimpanzee SFV; Genbank NC.001364.1); SFVpve-II SFV7 (genotype II Western chimpanzee SFV; Genbank ALJ11216.1); SFVptr-I Cam15 (genotype I Central chimpanzee SFV; Genbank ALJ11211.1); SFVptr-II huBAD327 (genotype II Central chimpanzee SFV; Genbank YP_009508547.1); SFVppy bella (Bornean orangutan SFV; Genbank YP_009508889.1); SFVhpi SAM106 (Pilated gibbon SFV; Genbank MF621235); SFVmfa Cy5061 (Crab-eating macaque SFV; Genbank BAU59306.1); SFVmcy-I FV21 (genotype I Taiwanese macaque SFV; Genbank QKY74209.1); SFVmcy-II_SFV2 (genotype II Taiwanese macaque SFV; Genbank AGM61337.1); SFVmmu-I DPZ9524 (genotype I Rhesus macaque SFV; Genbank MG051205.1); SFVmmu-II R289HybAGM (genotype II Rhesus macaque SFV; Genbank AFA44810.1); SFVmfu JM356 (Japanese macaque SFV; Genbank YP_009508557.1); SFVcni-II AG16 (genotype II Guenon SFV; Genbank AFX98100.1); SFVcae-I hu501 (genotype I African green monkey SFV; Genbank XDL44836.1); SFVcae-II LK-3 (genotype II African green monkey SFV; Genbank YP_001956723.2); SFVpan V909/03F (Olive baboon SFV; Genbank AZS32943.1); SFVpxx hu9406 (baboon spp SFV; Genbank AXY87476.1); SFVmsp-I MSP38 (genotype I mandrill SFV; Genbank QFG58495.1); SFVmsp-II MSP100 (genotype II mandrill SFV; Genbank MK014759.1); SFVcja mar (White-tufted-ear marmoset SFV; Genbank YP_009508578.1); SFVsxa Z17 (Yellow-breasted capuchin SFV; Genbank YP_009508583.1); SFVaxx VR-940 (Spider monkey SFV; Genbank YP_009508562.1); SFVbar (Southern woolly spider monkey SFV; Genbank AWO77077.1); SFVscc 1224 (Squirrel monkey SFV; Genbank YP_009508567.1); SFVocr PSFVgal (Brown greater galago proSFV; Genbank YP_009508542.1), FFVfca-I (genotype I feline FV; Genbank AAC58532.1); FFVfca-II (genotype I feline FV; Genbank CAA11582.1); BFVbta (bovine FV; Genbank AFR79245.1); EFVeca (equine FV; Genbank NP_054717.1).

### Statistics

All the statistical analyses were performed using GraphPad Prism 10. The numbers of experimental replicates and information on the statistical methods used for determination of two-tailed *p*-values are described in the individual figure legends. Symbols represent: *p < 0.05; **p < 0.01; ***p < 0.001; ****p < 0.0001; ns: not significant (*p* ≥ 0.05).

## Supplementary Information

Additional file 1.

Additional file 2: Figure S1. Physical particle release of various PFV GPC mutant containing vector supernatants based on nucleic acid composition. Cell-free viral supernatants harboring different GPs as indicated, or control supernatants harboring wt GP and enzymatically inactive RT (wt + iRT), containing no GP (VLP), or coming from a mock transfection with pUC19 (mock), were generated by transient transfection using a 4-component PFV vector system and virus particles concentrated by ultracentrifugation. Viral RNA (vRNA) and viral DNA (vDNA) content was determined by qPCR using PFV-specific primer-probe sets. Shown are Mean ±SD (n=2-8) of vRNA (A) or vDNA (B) content relative to wt PFV GP (wt). Figure S2. Infectivity of various PFV GPC mutant containing vector supernatants on different target cells. Cell-free viral supernatants, generated by transient transfection using a 4-component PFV vector system, and harboring different GPs as indicated, or control supernatants harboring wt GP and enzymatically inactive RT (wt + iRT), containing no GP (VLP), or coming from a mock transfection with pUC19 (mock), were titrated on different target cells as indicated. Viral titers were calculated from the flow cytometric determination of GFP expression 72 h.p.i.. vDNA content was determined by qPCR from pelleted viral particle nucleic acid extract. Except for the ctrls (wt+iRT, VLP, mock) relative infectivity values were normalized for physical particle (vDNA) content in comparison to the wt PFV GP (wt) sample. Peripheral residue GP mutants are indicated in light blue, central GP residue mutants in dark blue. Shown are Mean ±SD (n=5-8) of relative infectivity compared to wt PFV GP (wt) containing supernatants on the respective cell line. ns non-significant, * p<0.05, ** p<0.01, *** p<0.001, **** p<0.0001, by ordinary two-way ANOVA followed by Tukey’s multiple comparison test. Figure S3. Fluorescent binding assay for analysis of vector particle target cell binding. Plain, cell-free viral supernatants, generated by transient transfection using a 4-component PFV vector system, and harboring GFP-tagged PFV Gag in combination with different GPs as indicated, or control supernatants containing no GP (VLP) were incubated with different target cells and analyzed by flow cytometry as described in material and methods. (A) Top: A three-fold dilution series of wildtype PFV GPC containing supernatant was used to generated a standard curve for calculation of relative binding capacities of individual mutant GPC containing vector supernatants. Bottom: Examples of the GFP mean fluorescence intensity (GFP MFI) profiles of individual vector supernatants samples from a representative experiment on HT1080 target cells. (B) Summary of relative binding efficiencies of individual PFV GPC containing or control vector supernatants on HT1080 (dark green), Mouse L929 (green), and Sog9 (light green) target cells. Shown are Mean ±SD (n=2-4) of two independent vector supernatant productions. ns non-significant, * p<0.05, ** p<0.01, *** p<0.001, **** p<0.0001, by ordinary two-way ANOVA followed by Tukey’s multiple comparison test. Figure S4. Functional comparison of PFV and SFVmcy-I GPC central PCSP mutants. Cell-free viral supernatants, generated by transient transfection using a 4-component PFV vector system, and harboring different GP variants of PFV and SFVmcy-I as indicated, or control supernatants harboring the respective wt GP and enzymatically inactive RT (wt + iRT), containing no GP (VLP), or coming from a mock transfection with pUC19 (mock), were titrated on HT1080 target cells. Viral titers were calculated from the flow cytometric determination of GFP expression 72 h.p.i.. vDNA content was determined by qPCR from pelleted viral particle nucleic acid extract. Except for the ctrls (wt+iRT, no GP, mock) relative infectivity values were normalized for physical particle (vDNA) content in comparison to the respective wt PFV GP (wt) sample. The central residue GP mutants of PFV and SFVmcy-I are indicated in different shades of dark blue. Shown are Mean ±SD (n=4-7) of relative infectivity compared to wt PFV GP (PFV wt) containing supernatants generated in four independent vector productions on HT1080 target cells. **** p<0.0001, by ordinary two-way ANOVA followed by Tukey’s multiple comparison test. Table S1. qPCR primer.

## Data Availability

Data is provided with the manuscript or supplementary information files.
