# Supplementary figures and images for: Functional dissection of the prototype foamy virus glycoprotein heparan sulfate binding site
Source: Retrovirology. 2026 Mar 21;23:7. doi: 10.1186/s12977-026-00676-7 (PMC13064090; doi:10.1186/s12977-026-00676-7)

Fig 3A

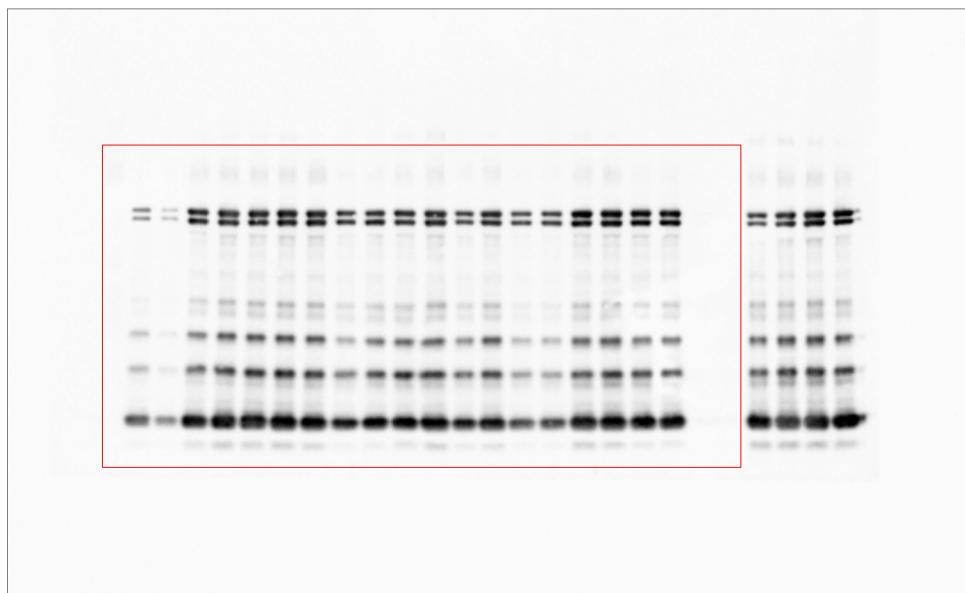

Fig 3B

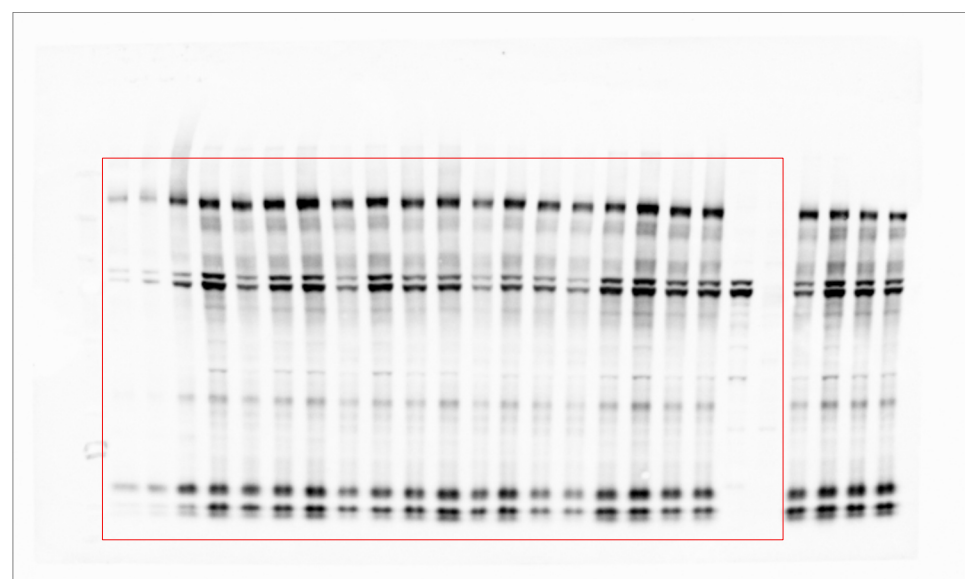

Fig 3C

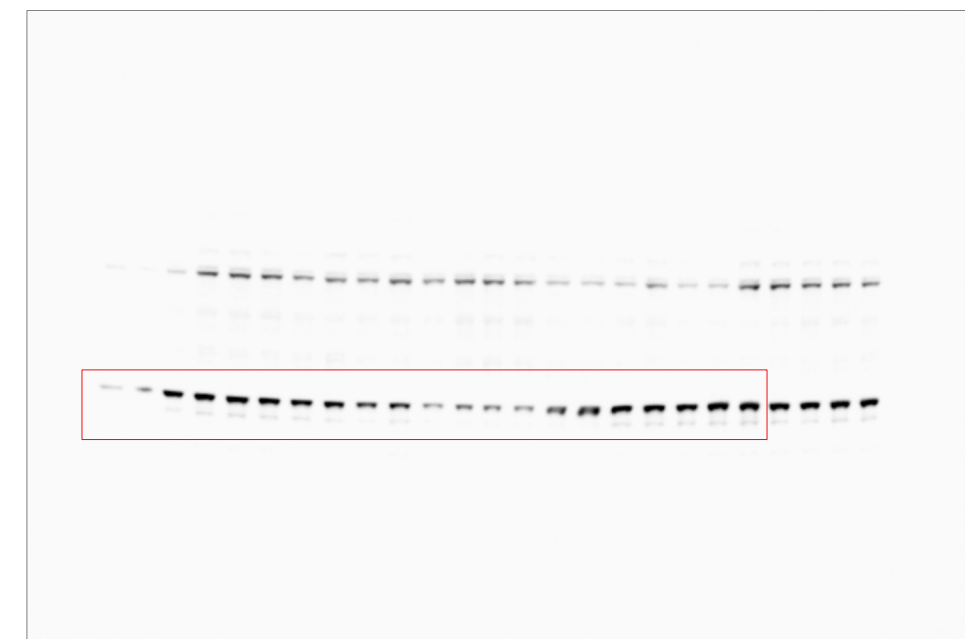

Supplement: Supplementary file 1 — Additional file 1. [file 12977_2026_676_MOESM1_ESM.pdf]
