# Supplementary material for: Functional dissection of the prototype foamy virus glycoprotein heparan sulfate binding site
Source: Retrovirology. 2026 Mar 21;23:7. doi: 10.1186/s12977-026-00676-7 (PMC13064090; doi:10.1186/s12977-026-00676-7)
Supplement: Supplementary file 2 — Additional file 2: Figure S1. Physical particle release of various PFV GPC mutant containing vector supernatants based on nucleic acid composition. Cell-free viral supernatants harboring different GPs as indicated, or control supernatants harboring wt GP and enzymatically inactive RT (wt + iRT), containing no GP (VLP), or coming from a mock transfection with pUC19 (mock), were generated by transient transfection using a 4-component PFV vector system and virus particles concentrated by ultracentrifugation. Viral RNA (vRNA) and viral DNA (vDNA) content was determined by qPCR using PFV-specific primer-probe sets. Shown are Mean ±SD (n=2-8) of vRNA (A) or vDNA (B) content relative to wt PFV GP (wt). Figure S2. Infectivity of various PFV GPC mutant containing vector supernatants on different target cells. Cell-free viral supernatants, generated by transient transfection using a 4-component PFV vector system, and harboring different GPs as indicated, or control supernatants harboring wt GP and enzymatically inactive RT (wt + iRT), containing no GP (VLP), or coming from a mock transfection with pUC19 (mock), were titrated on different target cells as indicated. Viral titers were calculated from the flow cytometric determination of GFP expression 72 h.p.i.. vDNA content was determined by qPCR from pelleted viral particle nucleic acid extract. Except for the ctrls (wt+iRT, VLP, mock) relative infectivity values were normalized for physical particle (vDNA) content in comparison to the wt PFV GP (wt) sample. Peripheral residue GP mutants are indicated in light blue, central GP residue mutants in dark blue. Shown are Mean ±SD (n=5-8) of relative infectivity compared to wt PFV GP (wt) containing supernatants on the respective cell line. ns non-significant, * p<0.05, ** p<0.01, *** p<0.001, **** p<0.0001, by ordinary two-way ANOVA followed by Tukey’s multiple comparison test. Figure S3. Fluorescent binding assay for analysis of vector particle target cell binding. Plain, cell-fre [file 12977_2026_676_MOESM2_ESM.pdf]

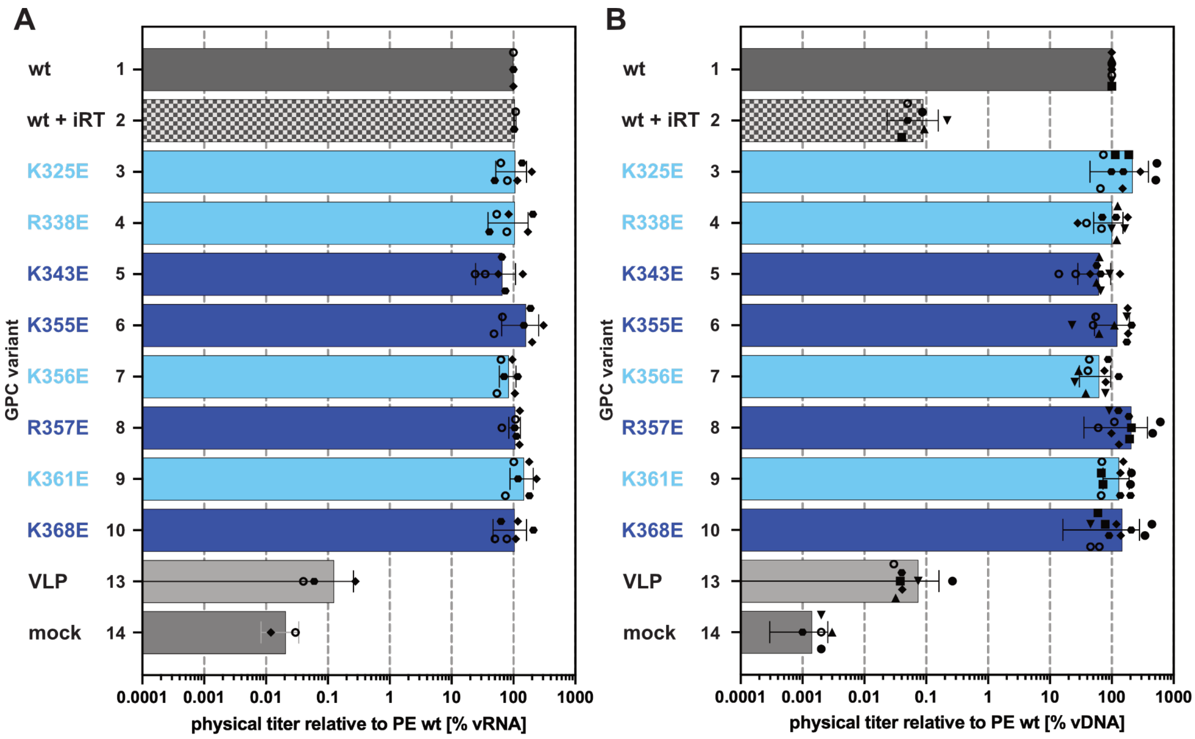

Figure S1 Physical particle release of various PFV GPC mutant containing vector supernatants based on nucleic acid composition.

Cell-free viral supernatants harboring different GPs as indicated, or control supernatants harboring wt GP and enzymatically inactive RT (wt + iRT), containing no GP (VLP), or coming from a mock transfection with pUC19 (mock), were generated by transient transfection using a 4-component PFV vector system and virus particles concentrated by ultracentrifugation. Viral RNA (vRNA) and viral DNA (vDNA) content was determined by qPCR using PFV-specific primer-probe sets. Shown are Mean ±SD (n=2-8) of vRNA (A) or vDNA (B) content relative to wt PFV GP (wt).

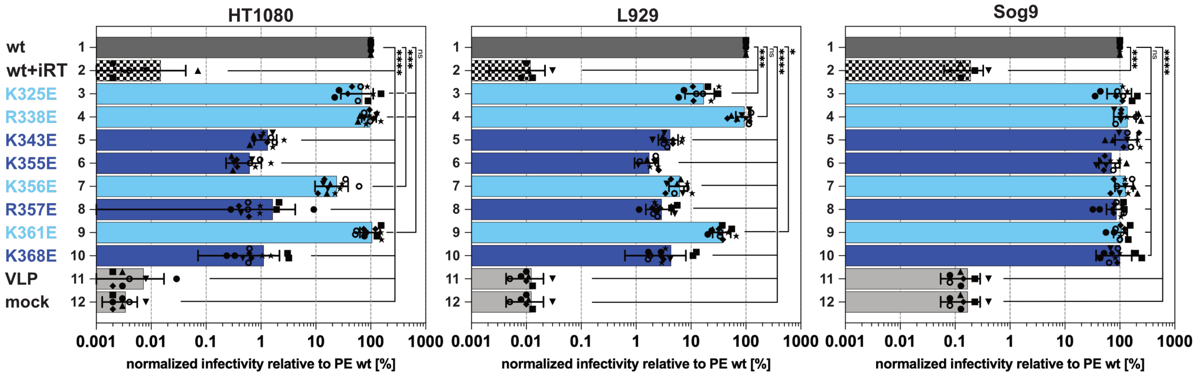

Figure S2 Infectivity of various PFV GPC mutant containing vector supernatants on different target cells.
Cell-free viral supernatants, generated by transient transfection using a 4-component PFV vector system, and harboring different GPs as indicated, or control supernatants harboring wt GP and enzymatically inactive RT (wt + iRT), containing no GP (VLP), or coming from a mock transfection with pUC19 (mock), were titrated on different target cells as indicated. Viral titers were calculated from the flow cytometric determination of GFP expression 72 h.p.i.. vDNA content was determined by qPCR from pelleted viral particle nucleic acid extract. Except for the ctrls (wt+iRT, VLP, mock) relative infectivity values were normalized for physical particle (vDNA) content in comparison to the wt PFV GP (wt) sample. Peripheral residue GP mutants are indicated in light blue, central GP residue mutants in dark blue. Shown are Mean ±SD (n=5-8) of relative infectivity compared to wt PFV GP (wt) containing supernatants on the respective cell line. ns non-significant, * $p<0.05$, ** $p<0.01$, *** $p<0.001$, **** $p<0.0001$, by ordinary two-way ANOVA followed by Tukey's multiple comparison test.

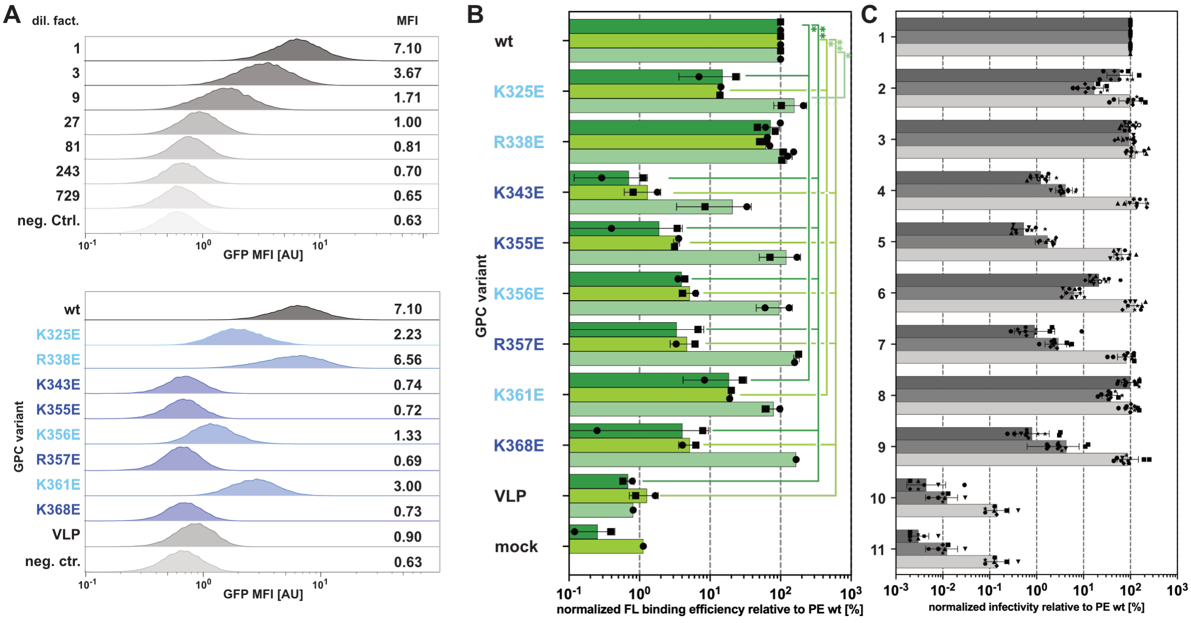

Figure S3 **Fluorescent binding assay for analysis of vector particle target cell binding.**
Plain, cell-free viral supernatants, generated by transient transfection using a 4-component PFV vector system, and harboring GFP-tagged PFV Gag in combination with different GPs as indicated, or control supernatants containing no GP (VLP) were incubated with different target cells and analyzed by flow cytometry as described in material and methods. **(A)** Top: A three-fold dilution series of wildtype PFV GPC containing supernatant was used to generated a standard curve for calculation of relative binding capacities of individual mutant GPC containing vector supernatants. Bottom: Examples of the GFP mean fluorescence intensity (GFP MFI) profiles of individual vector supernatants samples from a representative experiment on HT1080 target cells. **(B)** Summary of relative binding efficiencies of individual PFV GPC containing or control vector supernatants on HT1080 (dark green), Mouse L929 (green), and Sog9 (light green) target cells. Shown are Mean ±SD (n=2-4) of two independent vector supernatant productions. ns non-significant, * $p<0.05$, ** $p<0.01$, *** $p<0.001$, **** $p<0.0001$, by ordinary two-way ANOVA followed by Tukey's multiple comparison test.

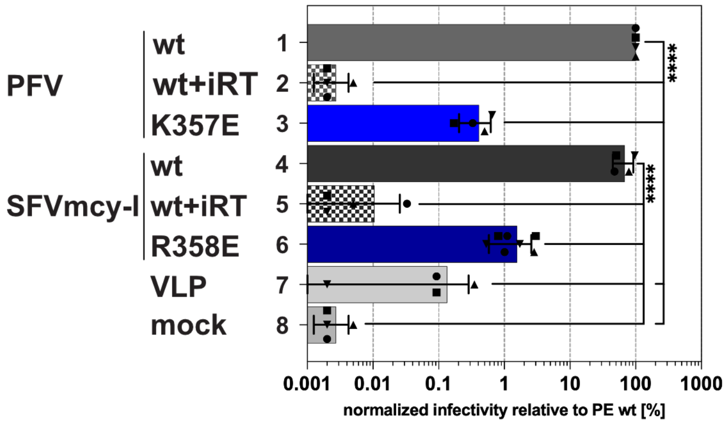

Figure S4 Comparison of PFV and SFVmcy-I GPC variants.

Cell-free viral supernatants, generated by transient transfection using a 4-component PFV vector system, and harboring different GP variants of PFV and SFVmcy-I as indicated, or control supernatants harboring the respective wt GP and enzymatically inactive RT (wt + iRT), containing no GP (VLP), or coming from a mock transfection with pUC19 (mock), were titrated on HT1080 target cells. Viral titers were calculated from the flow cytometric determination of GFP expression 72 h.p.i.. vDNA content was determined by qPCR from pelleted viral particle nucleic acid extract. Except for the ctrls (wt+iRT, no GP, mock) relative infectivity values were normalized for physical particle (vDNA) content in comparison to the respective wt PFV GP (wt) sample. The central residue GP mutants of PFV and SFVmcy-I are indicated in different shades of dark blue. Shown are Mean ±SD (n=4-7) of relative infectivity compared to wt PFV GP (PFV wt) containing supernatants generated in four independent vector productions on HT1080 target cells. **** p<0.0001, by ordinary two-way ANOVA followed by Tukey's multiple comparison test.

Table S1 qPCR primer

ID	Sequence 5' – 3'	Comment
3519	TGGACTTCGAGCAAGAGATG	ACTB qPCR fwd primer
3520	GAAGGAAGGCTGGAAGAGTG	ACTB qPCR rev primer
3521	CGGCTGCTTCCAGCTCCTCC	ACTB qPCR probe FAM/BHQ1
3821	AGGGAGACATCTAGTGATATAAG	PFV U5-Psi qPCR fwd primer
5186	TTATATGCCTCCCGCTATG	PFV U5-Psi qPCR rev primer
5187	ATTCCATGACAATTGGCGCCCAAC	PFV U5-Psi qPCR probe ATTO647/BHQ2
